# Pulmonary involvement in primary Sjögren's syndrome: contribution of the different means in the systematic investigation

**DOI:** 10.11604/pamj.2023.46.42.36534

**Published:** 2023-09-28

**Authors:** Maysam Jridi, Yosra Cherif, Samar Derbal, Fatma Ben Dahmen, Meya Abdallah

**Affiliations:** 1 Ben Arous Regional Hospital, Ben Arous, Tunisia,; 2 University of Tunis El Manar, Faculty of Medicine of Tunis, Tunis, Tunisia

**Keywords:** Sjögren's syndrome, interstitial lung disease

## Abstract

The prevalence of pulmonary involvement in primary Sjögren´s syndrome (pSS) varies depending on investigation methods. Our study aimed to identify the contribution of the different means of investigation in the systematic screening for pulmonary involvement in pSS. This is a retrospective and descriptive study including medical records of pSS patients, who validated the 2016 American College of Rheumatology/European League Against Rheumatism classification criteria for pSS and who had undergone pulmonary assessment. We enrolled 30 patients: twenty-nine females (97%) and one male (3%). The mean age was 55±17.4. In nine patients (30%), pulmonary disease revealed the diagnosis. Dyspnoea and cough were respectively reported by 43% (N=13) and 30% (N=9) of patients. The six-minute walk test showed desaturation in four cases (14%) and the percent predicted distance was less than 70% in three cases (11%). Pulmonary function tests (PFTs) showed restrictive patterns (N=7, 26%), obstructive patterns (N=2, 7%), combined patterns (N=1, 4%), and isolated small airway disease (N=1, 4%). The prevalence of pulmonary involvement based on high-resolution computed tomography (HRCT) was 31% (N=9/29). The most frequent interstitial lung disease (ILD) was observed in five scans (56%) and usual interstitial pneumonia was the most frequently seen in three cases (60%). Bronchiolitis was observed in four cases (25%) out of 16 abnormal scans. The six-minute walk test results correlated with PFT results (P<0.05). Pulmonary involvement was noted in 50% of cases (N=15). It was clinically silent in one-third of cases (N=5). In conclusion, pulmonary involvement in Sjögren´s syndrome can be asymptomatic in 33% of cases. The six-minute walk test has a strong correlation with the results of PFT, it should be considered as an assessment tool that reflects the functional state of the patient.

## Introduction

Primary Sjögren´s syndrome (pSS) is an autoimmune disease characterized by lymphocytic infiltration of exocrine glands responsible for sicca syndrome as well as extra glandular impairment and serological abnormalities. Primary Sjögren´s syndrome is a multi-system disorder and may result in several organ manifestations such as pulmonary manifestations which include airway abnormalities, interstitial lung disease (ILD), and lymphoproliferative disorders. Interstitial pneumonia and airway abnormalities often coexist [1].

Whether it is present at the initial assessment or a complication of the disease, the prevalence of pulmonary involvement in pSS is variable in different series depending on investigation methods and whether or not patients are symptomatic. When investigated systematically, the prevalence of pulmonary involvement in pSS increases to between 43% and 75% [1]. Chest imaging and pulmonary function tests are the principal means of diagnosis. Early detection of pulmonary involvement is crucial for improving pulmonary function and quality of life in pSS patients [2].

Screening for pulmonary involvement in Sjögren´s syndrome remains a topic of debate [2]. Systematic screening at the initial assessment for symptomatic and asymptomatic patients using clinical, radiological and spirometry means to detect and follow up the evolution of airways and parenchymal involvement has not been consensually approved.

Our study aimed to identify the contribution of the different means of investigation: clinical, chest radiography, computed tomography and pulmonary function tests in the systematic screening for pulmonary involvement in pSS.

## Methods

**Study design and setting:** this is a retrospective and descriptive study, conducted in the Internal Medicine Department of Ben Arous Regional Hospital from March 2015 to March 2020.

**Study population:** the study targeted adult patients with primary Sjögren´s syndrome. The inclusion criteria were: age ≥ 18 years old, patients who validated the 2016 American College of Rheumatology/European League Against Rheumatism (ACR/EULAR) classification criteria for primary Sjögren´s syndrome and had at least one chest tomography and/or spirometry. Patients with Sjögren´s syndrome associated with other connective tissue diseases or autoimmune diseases were not included. Patients who had no pulmonary investigation in their medical files, those who did not show up for check-ups for more than two years, and patients with medical history of other chronic pulmonary diseases (chronic obstructive pulmonary disease (COPD) or asthma) were excluded.

**Data collection:** we used the electronic health records system in the hospital to generate a preliminary list of Sjögren´s syndrome medical files using the code M35.0 corresponding to “sicca syndrome [Sjögren]” according to the international classification of diseases 10^th^ revision. We extracted 170 medical records. Then we went through each medical record and applied the inclusion and exclusion criteria in order to extract the target population. Afterwards, we collected: demographic data, history of exposure to tobacco or tandoor smoke and airborne allergens, subjective and objective sicca signs and the duration of symptoms, the six-minute walk test results [3,4], pulmonary function tests results, chest X-rays and high-resolution computed tomography results.

**Definitions:** pulmonary involvement was defined as typical HRCT abnormalities including the presence of bronchiolitis or ILD and excluding non-specific lesions and/or PFT abnormalities. Pulmonary disease onset pSS was defined by patients who were referred to the internal medicine ward by pneumologists due to the presence of respiratory complaints or abnormal HRCT imaging before the diagnosis of pSS.

**Statistical analysis:** IBM SPSS Statistics® version 25 was used. In order to distinguish the normally distributed groups from the non-normal ones, we used both Kolmogorov-Smirnov and Shapiro-Wilk tests associated with their skewness and kurtosis. Then, normally distributed variables were expressed as the mean ± standard deviation, while the non-normally distributed variables were expressed as the median and the interquartile range. Categorical data were expressed as the number of patients (N) and the relative percentage (%). We used the Chi-squared test for categorical data. For contingency tables, we used Fisher´s exact test. Regarding continuous data, we deployed the Student T-test when comparing normally distributed groups, the Mann-Whitney non-parametric test for non-normal groups, while we used Welch and Brown-Forsythe robust t-tests to compare groups with unequal variances and unequal sample sizes or in case of heteroscedasticity. Statistical significance was set at the p ≤ 0.05 level. We used a 95% confidence interval.

**Ethical considerations:** patients´ data were used anonymously; no participation consent was needed. Consent of the ethics committee of the hospital was granted.

## Results

### Descriptive analysis

**Clinical presentation:** the study included 30 patients. The gender ratio (female/male) was 29: 1. The age at disease onset ranged between 25 and 93 and the mean age was 53±17.4 history of exposure revealed that only one patient was an ex-smoker and that none had a professional exposure to silicone, birds or other airborne allergens. Three patients admitted tobacco snuffing and five reported exposures to tandoor smoke in their twenties that ended at least ten years before the diagnosis. Twenty-nine patients (97%) reported sicca symptoms before the establishment of the diagnosis and the mean duration of symptoms was 22 months (2-48). Pulmonary manifestations revealed the disease in nine patients. Sicca syndrome characteristics are illustrated in Table 1.

**Table 1:** sicca syndrome characteristics

Sicca syndrome	Patients (N)
Xerophthalmia	25
Xerostomia	27
Ophthalmic exams (N=16): altered BUT	10
KCS	5
Schirmer’s test: ≤5 mm/5 minutes in at least one eye	20
Unstimulated salivary flow rate ≤ 1.5 mL/15 minutes	13
Salivary gland biopsy results: focus score ≥ 1 foci /4 mm^2^	27

BUT: break up time; KCS: keratoconjunctivitis sicca

**Figure 1:**
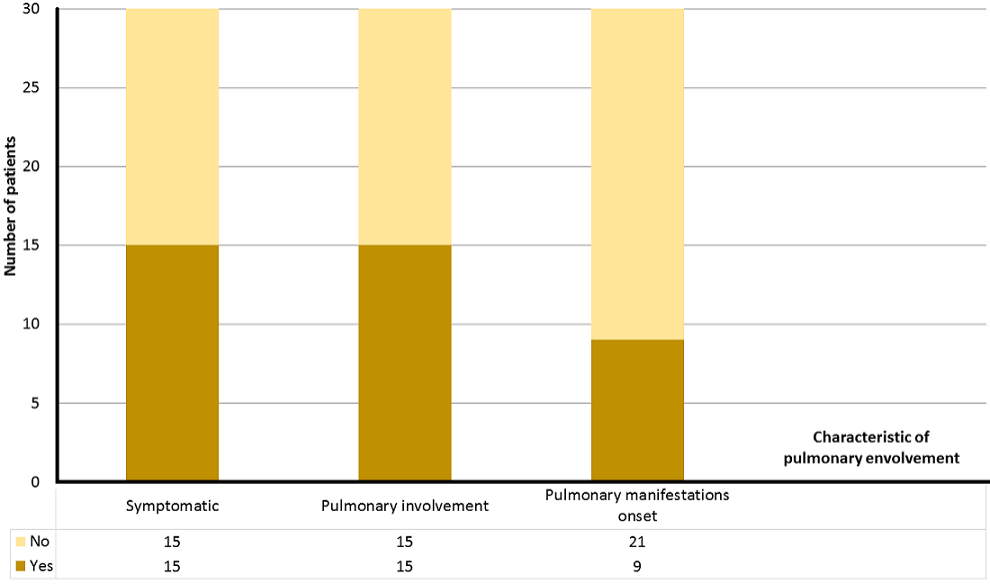
splitting the study population of primary Sjögren’s syndrome patients according to symptoms, pulmonary involvement and pulmonary disease onset

**Pulmonary assessment:** fifteen patients (50%) were symptomatic: breathlessness was reported by thirteen patients (43%) and dry cough was signaled by nine (30%). The physical exam revealed normal auscultation sounds in 22 cases (73%) while seven (23%) had crackles and one (3%) had Ronchi. The six-minute walk test was conducted for 28 patients (93%). The mean percent predicted six-minute walk distance was 86% (30-105), it was under 70% in three cases (11%). During the test four patients (14%) presented desaturation and ten (36%) reported dyspnoea. Table 2 illustrates the results of the conducted six-minute walk tests, had pulmonary function tests (PFTs) were performed in twenty-seven cases (90%) and showed that 16 patients (59%) had normal PFTs, while seven (26%) had restrictive patterns, two (7%) had non-reversible obstruction, one patient (4%) had combined restrictive and obstructive pattern and another one (4%) had isolated small airway disease. Chest radiographs were systematically conducted: 18 (60%) were normal while 12 (40%) showed abnormalities: interstitial pattern (N=9, 30%), opacities (N=2, 7%), bronchial pattern (N=1, 3%). Twenty-nine patients had chest computed tomography, among them 55% (N=16) showed abnormalities illustrated in Table 3 and Table 4. Lung involvement based on HRCT findings alone was noted in 31% (9/29) of cases: distributed as interstitial lung disease (ILD) in five cases (56%), and as signs of airway disease in four patients (44%). Usual interstitial pneumonia (UIP) represented 60% (N=3) of ILD patterns. It was characterized by honeycombing, reticular pattern, bronchial wall thickening, septal thickening, and bronchiectasis. On the other hand, non-specific interstitial pneumonia (NSIP) was noted in one patient (20%). High-resolution computed tomography abnormalities of this type were a ground-glass pattern, septal thickening, reticular patterns, airspace consolidation, and nodules. Lastly, one patient (20%) had lymphocytic interstitial pneumonia (LIP), characterized by: ground-glass attenuation, septal thickening, reticular pattern, bronchiectasis, lung cysts, and vascular bundle thickening. Fifteen patients (50%) had pulmonary involvement based on functional tests and imaging. Pulmonary disease revealed the diagnosis of pSS in 30% (N) of cases (Figure 1).

**Table 2:** six-minute walk test results

Percent predicted walk distance		Dyspnoea		Desaturation	
	Number of patients	Yes	No	Yes	No
30	1	1	-	1	-
68	1	-	1	-	1
69	1	-	1	-	1
80	1	1	-	-	1
82	2	-	2	-	2
83	1	-	1	-	1
86	1	1	-	-	1
88	2	1	1	-	2
90	3	2	1	-	3
95	3	-	3	-	3
98	1	1	-	-	1
100	10	2	8	2	8
105	1	1	-	1	-
Total N (%)	28 (100%)	10 (36%)	18 (64%)	4 (14%)	24 (86%)

**Table 3:** high resolution computed tomography abnormalities in order of frequency

HRCT findings	N
Nodules	10
Bronchiectasis	6
Ventilatory disorder	6
Airspace consolidation	5
Distortion	5
Reticular pattern	4
Septal thickening	4
Ground-glass attenuation	3
Linear opacities	3
Emphysema	3
Mediastinal lymph nodes	3
Honeycombing	2
Mosaic perfusion	2
Bronchial wall thickening	2
Tree in bud	2
Lung cysts	2
Bronchiolectasis	2
Vascular bundle thickening	1
Total	16 abnormal HRCTs

HRCTs: high-resolution computed tomographys

**Table 4:** high resolution computed tomography patterns

Diagnosis	Number of patients
Normal	13
Non-specific lesions	7
Bronchiolitis	4
UIP	3
NSIP	1
LIP	1

UIP: usual interstitial pneumonia; NSIP: non-specific interstitial pneumonia; LIP: lymphocytic interstitial pneumonia

### Analytic analysis

**Comparing symptomatic and asymptomatic cases:** one third of the whole cohort (10/30) were symptomatic and had objective pulmonary involvement. In the other hand, it was clinically silent in one-third of asymptomatic cases (5/15) (Table 5).

**Table 5:** comparing symptomatic and asymptomatic primary Sjögren’s patients

Comparison parameters	Symptomatic	Asymptomatic	P
Age (years)^a^	58.5 (±18.7)	51.3 (±15.6)	NS
Duration of sicca symptoms before diagnosis (months)^a^	21.9 (±14.7)	22.5 (±14)	NS
Subjective xerophthalmia (number of patients)^b^	12	13	NS
Subjective xerostomia (number of patients)^b^	15	12	NS
Salivary flow (ml)^a^	3.3 (±3.8)	2.9 (±2.2)	NS
Labial biopsy: Chisholm stage^a^	4 (±1)	3 (±1)	0.010*
Schirmer’s test (mm)^a^	N=15	N=15	
Right eye	14 (±11.9)	7.6 (±7.4)	0.027*
Left eye	11.9 (±11.8)	5.4 (±3.7)	0.001**
ESSDAI score^a^	12.6 (±4.8)	7 (±5.3)	0.049*
Six-minute walk test:	N=14	N=14	
Percent predicted distance^a^	87.6% (±19.1)	92% (±10)	NS
Desaturation^b^	4	0	0.037*
PFT findings:	N=14	N=13	
Normal^b^	7	9	NS
Restrictive pattern^b^	5	2	NS
Obstruction^b^	1	1	NS
Combined restrictive and obstructive pattern^b^	1	0	NS
Isolated small airway disease^b^	0	1	NS
**Chest X-rays**			
Normal^b^	5	13	0.003**
HRCT findings	N=14	N=15	
Normal^b^	4	9	NS
Non-specific lesions^b^	2	5	NS
Bronchiolitis^b^	3	1	NS
UIP^b^	3	0	0.050*
LIP^b^	1	0	NS
NSIP^b^	1	0	NS
Pulmonary involvement^b^	10	5	0.048*
ANA positivity^b^	6/15	5/14	NS
ANA title^a^	1032 (±865)	302 (±280)	0.015*
SSA^b^	6/15	5/14	NS
SSB^b^	1/15	1/14	NS
RF positivity^b^	1/8	7/13	0.050*
RF abnormal Value^a^	51.5	69.4 (±80)	NS

ESSDAI: EULAR Sjögren's syndrome disease activity index; NS: non-significant; PFT: pulmonary function test; HRCT: high resolution computed tomography; UIP: usual interstitial pneumonia; LIP: lymphocytic interstitial pneumonia; NSIP: non-specific interstitial pneumonia; ANA: anti-nuclear antibodies; SSA: anti-sicca syndrome A; SSB: anti-sicca syndrome B; RF: rheumatoid factor; NS: non-significant; * p < 0.05; **p < 0.005; ^a^: normally distributed variables were expressed as the mean ± standard deviation, while the non-normally distributed variables were expressed as the median and the interquartile range; ^b^: categorical data were expressed as the number of patients (N=)

**Comparing patients with pulmonary involvement and patients without pulmonary involvement:** the only significant difference between these two groups was more altered Schirmer´s test results in patients with no pulmonary involvement. Patients with pulmonary involvement had a slightly higher frequency of dyspnoea and four (27%) of them presented desaturation in the six-minute walk test compared to no patient in the other group.

**Comparing pulmonary disease onset pSS with no pulmonary disease onset pSS:** among symptomatic patients (N=15), nine (60%) had pulmonary manifestations revealing pSS. Pulmonary manifestations revealing pSS were significantly more prevalent in older patients (Table 6). Patients with interstitial lung disease had pulmonary disease as the first manifestation of pSS (5/30) representing 17% of the cohort.

**Table 6:** comparing pulmonary disease onset primary Sjögren’s syndrome (pSS) with no pulmonary disease onset pSS

Comparison parameters	Pulmonary manifestations revealing pSS		p
	Yes N=9	No N=21	
Age (years)^a^	66 (±19)	50 (±15)	0.019*
Duration of sicca symptoms before diagnosis (months)^a^	21 (±17)	23 (±13)	NS
Subjective xerophthalmia (number of patients)^b^	8	17	NS
Subjective xerostomia (number of patients)^b^	9	18	NS
Salivary flow (ml)a	1.9 (±0.5)	3.8 (±1.5)	0.024*
Labial biopsy: chisholm stage^a^	3.5 (±0.5)	3 (±1)	0.049*
**Schirmer’s test (mm) ^a^**			
Right eye	13 (±5.5)	10 (±4.5)	NS
Left eye	11.6 (±6)	8 (±5)	NS
Six-minute walk test:	(N=8)	(N=19)	
Percent predicted distance^a^	87.56% (±19.1)	92% (±10)	NS
Desaturation^b^	3	1	*0.031
**PFT findings:**	**(N=9)**	**(N=18)**	
Normal^b^	3	12	NS
Restrictive pattern^b^	4	3	NS
Obstruction^b^	1	1	NS
Combined restrictive and obstructive pattern^b^	1	0	NS
Isolated small airway disease^b^	0	1	NS
**Chest X-rays**			
Normal^b^	1	17	**0.001
**HRCT findings**	**(N=9)**	**(N=20)**	
Normalb	1	12	*0.018
Non-specific lesionsb	1	6	NS
Bronchiolitisb	2	2	NS
UIPb	3	0	*0.023
LIPb	1	0	NS
NSIPb	1	0	NS


PFT: pulmonary function test; HRCT: high resolution computed tomography; UIP: usual interstitial pneumonia; LIP: lymphocytic interstitial pneumonia; NSIP: non-specific interstitial pneumonia; NS: non-significant; * p < 0.05, **p < 0.005; ^a^: normally distributed variables were expressed as the mean ± standard deviation, while the non-normally distributed variables were expressed as the median and the interquartile range; ^b^: categorical data were expressed as the number of patients (N=)

**Correlating the six-minute walk test results to PFT findings:** correlating the six-minute walk test results to PFT findings showed that 93% (N=14/15) of normal spirometry corresponded to normal six-minute walk tests (P<0.05) (Table 7).

**Table 7:** pulmonary function test (PFT) patterns and the six-minute walk test results

PFT findings	Six-minute walk test		Total	Correlation
	Normal	Abnormal		
Normal				P < 0.05
N	14	1	15	
Abnormal				
N	5	4	9	
Total	19	5	24	

## Discussion

The current study aimed to identify the contribution of the different means of investigation: in the systematic screening for pulmonary involvement in pSS. Respiratory complaints related to pSS were described in 50% of the study group. Pulmonary involvement as defined by typical abnormalities in HRCT or impaired functional tests were found in 50% of the cohort: ten patients (67%) were symptomatic while five were asymptomatic (33%). Based on HRCT findings alone, lung involvement was noted in 31% (9/29) of cases: distributed as ILD in five scans (56%) and as signs of airway disease in four cases (44%). The most frequent pattern in this study was usual interstitial pneumonia. In this study the prevalence of ILD before pSS was 5/30 (17%). As for functional tests, four asymptomatic patients had abnormal PFT patterns. The restrictive pattern was the most frequent. A strong correlation was found between a normal six-minute walk test and a normal function assessment.

Respiratory complaints related to pSS were described in 50% of the study group compared to 21.5% in Kampolis *et al*. study [5], third of which were attributed to other lung diseases and comorbidities. Kelly *et al*. reported respiratory symptoms to be present in 43% of patients at baseline [6]. Unlike some studies [7], the predominant complaint in our patients was shortness of breath affecting 43% of patients, while dry cough was reported by 30% of them. However, our results are in line with Manuel Ramos-Casals *et al*. review [8], reporting dyspnoea in 62% of patients and dry cough in 54% of patients.

In the current study, 50% of the cohort, whether or not they were symptomatic, had pulmonary involvement as defined by typical abnormalities in HRCT or impaired functional tests. In Constantopoulos *et al*. study based on clinical, radiologic, functional, and histologic data lung involvement was reported to reach up to 75% of pSS patients [9]. Pulmonary involvement in the symptomatic group (N=15) was found in ten patients (67%) while it was reported in 59/216 (27%) in Palm *et al*. study of symptomatic cohort [10]. It is noteworthy that one-third (5/15) of the asymptomatic patients had pulmonary involvement: four of them had abnormal PFTs compared to 17/34 abnormal performed PFTs in Uffmann *et al*. study that included only asymptomatic patients [11]. In literature ILD has been reported to be diagnosed before pSS in one-fifth of cases (20%) [12], which is close to the result of this study (17%). Studies systematically performing HRCT exams in all patients with pSS have demonstrated lung involvement in up to 42% [13,14]. However, in our study it was noted in 31% (9/29) of cases: distributed as ILD in five scans (56%) and as signs of airway disease in four cases. The most frequent ILD type in this study was usual interstitial pneumonia unlike most studies [2,8,15-17]. Yet it corroborates the results of Sogkas *et al*. study conducted in 2020 reporting UIP to be the predominating pattern occurring in 42% of HRCT findings [7]. Due to the small study population, ILD results hardly reflect the different patterns shown in large cohort studies. Also, the difference in UIP prevalence compared to other studies reporting predominance of NSIP may be explained by the difficulty of the diagnosis of UIP and to its often coexistence with NSIP. Four asymptomatic patients had abnormal PFT patterns, suggesting that pulmonary function tests could be useful to identify sub-clinical types of lung involvement in pSS at an early stage of the disease as discussed in previous reviews [18]. The restrictive pattern was the most frequent among impaired functional tests in agreement with previous studies [8,17].

Annex 1 is a summary of the different means of investigation concerning pulmonary involvement in pSS patients as well as the conclusions that were reported in different studies and ours. It demonstrates how heterogeneous the concept of pulmonary evaluation is. Furthermore, it explains the differences in the prevalence of pulmonary involvement in pSS patients in different countries.

**Figure 2:**
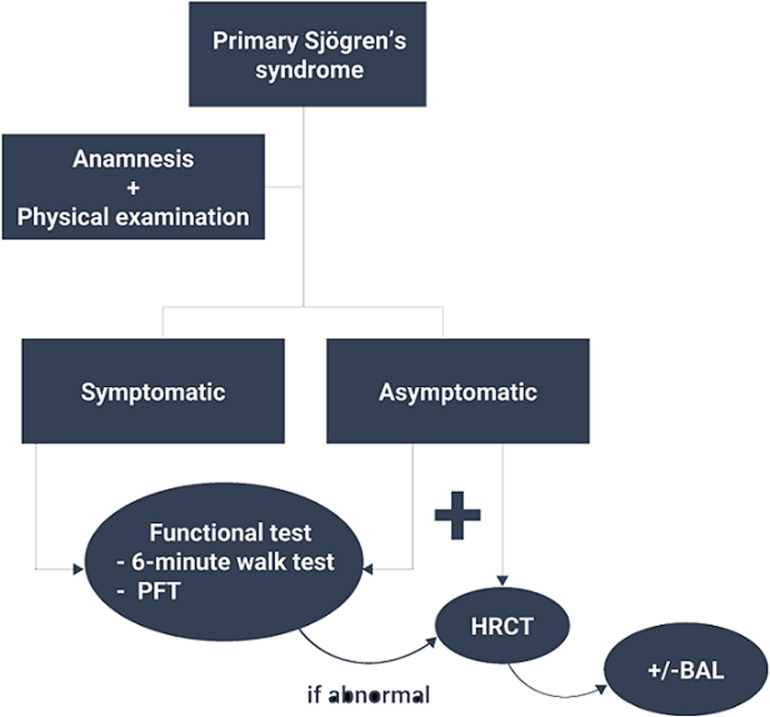
proposed recommendations’ algorithm for the systematic screening of pulmonary involvement in primary Sjögren’s syndrome patients

Based on the study results, HRCT and PFT should be performed when patients are symptomatic at onset. When patients are asymptomatic, we can start with the less invasive and easier tools such as the six-minute walk test which results showed an interesting correlation with PFT results. When abnormal, further investigations are needed. Abnormal PFT or HRCT findings in asymptomatic patients reflect subclinical pulmonary involvement. Hence, we suggest conducting a PFT at baseline in asymptomatic patients as a reference pulmonary assessment. HRCT findings in asymptomatic patients showed mostly non-specific lesions which can be either subtle lung changes or early lesions that might progress through the years. Nevertheless, HRCT is crucial to get a detailed description of ILD and airway involvement as well. HRCT has all its importance in defining the disease pattern and may be interesting in follow-ups after treatment. In light of the results of the previous explorations, patients will either need closer follow-ups, further investigations such as bronchoalveolar lavage or biopsies, or even treatment (Figure 2).

To our knowledge, this is the first study that used the six-minute walk test to assess pulmonary involvement in pSS patients. Its convenience as an evaluation tool for lung function and performance of daily living activities as well as predicting mortality has been previously reported in patients with idiopathic pulmonary fibrosis [19]. A limitation of the current study is the small population. In fact, this was due to many factors. Firstly, primary Sjögren´s syndrome itself is a rare disease [20]. Secondly, the study was monocentric. Thirdly, strict inclusion criteria: only pSS patients while secondary Sjögren´s syndrome cases were not included to accurately identify pulmonary involvement and avoid interference with other autoimmune diseases.

## Conclusion

The current study demonstrated that half of pSS patients presented some sort of respiratory complaints. Therefore, pulmonary anamnesis should be a routine systematic performance at baseline and during follow-ups. Lung involvement was clinically silent in 33% of cases and PFTs may be abnormal in asymptomatic patients. Thus, systematically performing PFT at baseline, is recommended. Since the six-minute walk test has a strong correlation with the results of PFT, it should be considered as an assessment tool that reflects the functional state of the patient. It hasn´t been used in previous studies concerning pSS lung disease. We believe it´s an effective low-cost means of exploration and it gives an idea about the quality of life as well. High-resolution computed tomography findings are in line with several studies and corroborate the fact that HRCT is a very sensitive means in the detection of parenchymal abnormalities that have a non-specific aspect. Hence, it can be required if symptoms occur. Pulmonary manifestations revealed pSS in 30% (9/30) of patients in our study group. Such patients are usually referred to internists after consulting other specialists, such as pneumologists. Therefore, systematically looking for sicca symptoms should be recommended in ILD patients.

### What is known about this topic

- *Pulmonary involvement in primary Sjögren´s syndrome is an important systemic domain affecting the quality of life;*
- *Different means of investigation are available;*
- *The systematic screening remained not well codified*.

### What this study adds

- *Pulmonary involvement in primary Sjögren´s syndrome can be clinically silent in 33% of patients, when systematically screened;*
- *The six-minute walk test correlates with pulmonary functions tests and should be performed systematically;*
- *Pulmonary involvement can reveal primary Sjögren´s syndrome in 30% of cases*.

## Annex

**Annex 1**: pulmonary involvement in primary Sjögren´s syndrome in different studies (PDF-101 Kb)
